# Clinical aspect, pathogenesis and therapy options of alopecia induced by hormonal therapy for breast cancer

**DOI:** 10.37349/etat.2021.00059

**Published:** 2021-10-31

**Authors:** Alfredo Rossi, Gemma Caro, Francesca Magri, Maria Caterina Fortuna, Marta Carlesimo

**Affiliations:** Department of Clinical Internal Anesthesiological and Cardiovascular Sciences, Sapienza University of Rome, 00161 Rome, Italy; University of Edinburgh, UK

**Keywords:** Alopecia, hormonal therapy, breast cancer

## Abstract

Adjuvant hormonal therapy is one of the most important treatments of hormone-receptor-positive breast cancer and includes selective estrogen receptor modulators, aromatase inhibitors, and luteinizing hormone-releasing hormone analogs. In patients receiving these drugs, a progressive recession of frontal-temporal hairlines is often observed, such as a certain grade of hair miniaturization in the same areas and the central scalp area, producing a pseudo-female androgenic alopecia, which has to be considered oncotherapy-induced alopecia. The aim of this work, is to describe the clinical aspects and pathogenesis of this type of alopecia and to analyze the different drugs which have been proposed until now. The authors concude that topical hormones should not be considered as a therapeutic approach because of their direct or indirect oncogenic potential. A therapeutic approach that could be both safe and effective is proposed.

## Introduction

Endocrine-based cancer therapies are widely used at various stages of treatment and prevention for many types of solid tumors [1].

Currently, adjuvant hormonal therapy (HT) is one of the most important treatments of hormone-receptor-positive breast cancer in terms of reduction of recurrence and mortality [2–3]. These therapies include selective estrogen receptor modulators (SERM), such as tamoxifen, aromatase inhibitors (AI), and luteinizing hormone-releasing hormone (LHRH) analog. The treatment is usually prescribed for 5 years or more [4].

Selective estrogen receptor degraders (SERD)/downregulators are now in research and development [5].

## Clinical aspect of alopecia induced by HT for breast cancer

The frequency and the clinical features of alopecia associated with cytotoxic chemotherapy are well-known, while the incidence of all-grade alopecia in patients receiving endocrine therapies is less frequently described. It has currently been reported to range from 0% to 25.4% [1].

Clinically, a progressive recession of frontal-temporal hairline is present, associated with a certain grade of hair miniaturization in the same area and also in the central area of the scalp [6–10]. It develops within 12 months of HT in most cases.

It is not severe alopecia, being reported as a grade 1 in the 93% of patients using common terminology criteria for adverse events, nevertheless, alopecia-related quality of life (QoL) is deeply affected. The mean score of the Hairdex questionnaire is reported to be 25.6, with a higher negative impact on emotions [11].

## Pathogenesis of alopecia induced by HT for breast cancer

The role of estrogens in modulating hair growth is well known [12], and aromatase has a protective role for anagen hair follicles of the frontal hairline.

Aromatase p450 (p450arom) is responsible for the conversion of testosterone to estradiol, with the result of reducing testosterone and dehydrotestosterone (DHT) levels [13]. Its inhibition is useful in all estrogen-responsive cancers. Aromatase is also localized in the inner root sheath of hair follicles, and in particular, it is most highly expressed at the frontal and occipital scalp areas of women than at the same areas of men [14].

**Figure 1.**
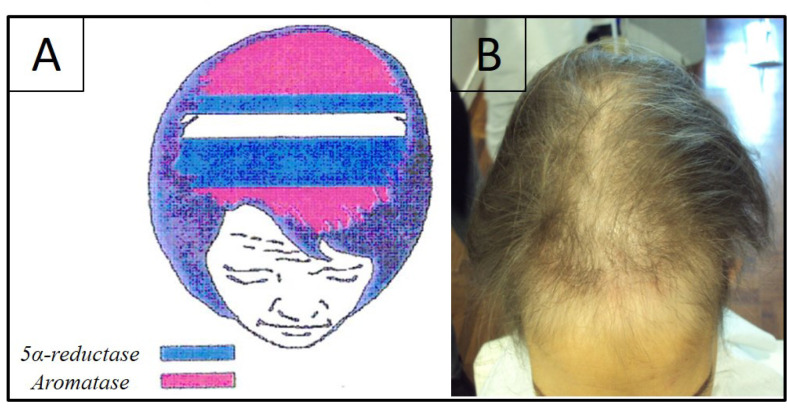
A) Representation of aromatase levels in a woman’s scalp. They are higher at the frontal and occipital scalp areas (pink), areas in blue represent the zones where 5α-reductase is higher; B) clinical aspect of alopecia induced by hormonal anticancer therapy. It shows the recession of the frontal-temporal hairline and a certain grade of hair miniaturization in the same area and also in the central scalp area

Blocking estrogen synthesis, AI cause a relative increase of 5α-reductase activity because of an upstream testosterone accumulation with the effect of higher production of DHT around hair follicles. This increase in DHT levels leads to a shorter anagen phase (which is the phase of hair growth), with a consequent male pattern of hair loss, mimicking female androgenetic alopecia (FAGA) which we call “pseudo-male pattern androgenetic alopecia (AGA)”. Sebaceous secretion also increases, with changes in the hair texture and appearance. It is important to clarify that it is not a FAGA, but it has to be considered oncotherapy-induced alopecia. Moreover, the lack of involvement of the anterior scalp area and vertex underlines the difference between this type of alopecia and a real FAGA [15] (Figure 1).

Concerning the other drugs employed in HT of breast cancer, they act at several levels and with several mechanisms (upstream blocking LHRH release, or downstream inhibiting estrogen receptors), but they all have the same final effect of inhibiting estrogenic activity. For this reason, their effects on hair follicles will be visible not only where aromatase is more represented, but also at the anterior scalp area and the vertex. Anyway, the clinical aspect is similar to the one caused by AI, so we finally observe a pseudo-FAGA.

No data are currently available about the hair loss associated with SERD treatment.

Herein we report and discuss the therapy options which have been proposed for the prevention and treatment of HT-induced alopecia and we give our therapeutic proposal.

## Therapy options of alopecia induced by HT for breast cancer

### Estrogens

Standing to the pathogenesis of this type of alopecia the most effective solution would be the topical application of estrogens. Even if reports about the use of these molecules exist, we do not recommend them because of their possible oncogenic effects if they enter the cardiovascular system and/or because they could favor skin metastasis when topically administered. It is important to remember that HT is administered in patients with hormone-sensitive breast cancer, and higher blood levels of estrogens could enhance tumor growth.

17β-estradiol is a hormone to all effects, able to bind estrogen receptors, so it should not be used in this kind of patient. 17α-estradiol is an isoform of 17β-estradiol which is incapable of binding estrogen receptor, and for this reason, it was considered suitable for the treatment of alopecia induced by HT [10]. It has been demonstrated that 17α-estradiol is able to increase testosterone transformation in 17β-estradiol and androstenedione in estrone, probably by increasing aromatase activity [16]. So even this isoform, although unable to bind hormonal receptors, could be indirectly dangerous.

### Anti-androgen molecules

**Figure 2.**
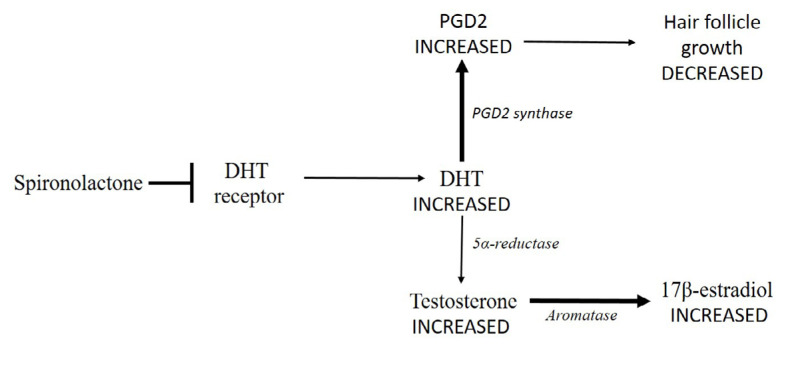
Spironolactone impedes DHT to bind its receptor (➡) leading to a DHT accumulation. Consequently, DHT transformation into testosterone is delayed (thinner arrow), so that testosterone concentration increases. Testosterone conversion into 17β-estradiol by aromatase could increase (thicker arrow). Moreover, high levels of DHT lead to an increased synthesis of PGD2 by PDG2 synthase (thicker arrow), which has a negative effect on hair follicle growth (thinner arrow)

Since it was clear that no estrogens could be employed, it was proposed to use anti-androgen molecules, such as spironolactone [10]. It competes for the binding site of the aldosterone receptor in the kidney, but it is also able to bind the DHT receptor so that it also has anti-androgen activity. Anyway, by binding the DHT receptor, spironolactone could activate a series of events that end with an increased estrogen production (Figure 2). Theoretically, spironolactone would increase DHT concentration with a consequently delayed transformation of testosterone, which would accumulate. Moreover, it has been demonstrated that high levels of DHT lead to an increased synthesis of prostaglandin D2 (PGD2), which has a negative effect on hair follicle growth [17] (Figure 2).

**Figure 3.**
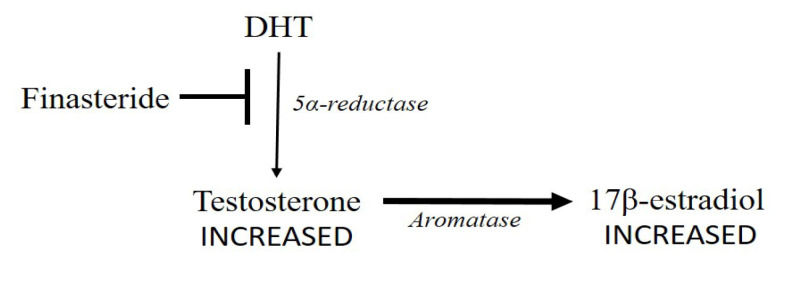
Finasteride blocks (➡) 5α-reductase (thinner arrow) leading to an increase in testosterone concentration. Testosterone conversion into 17β-estradiol by aromatase could increase (thicker arrow)

Finasteride has also been proposed [10], but even this molecule could provoke an increased estrogen production. The block of 5α-reductase would lead to a higher testosterone concentration (Figure 3).

An increased quantity of testosterone could have different effects depending on the HT agent in use. In the case of treatment with SERMs aromatase is able to transform testosterone to 17β-estradiol, and if its concentration grows, it could compete with SERM’s binding to the receptor. If the patient is taking LHRH analogs, the greater quantity of testosterone would be transformed to 17β-estradiol and it would bind its receptors if treatment with SERMs is not also given. In the case of the patient receiving AI, testosterone transformation into 17β-estradiol is theoretically blocked, but it cannot be excluded that testosterone could compete with binding to the enzyme and, having a better affinity for it, it could be transformed anyway.

For these reasons all the molecules which increase testosterone concentration (spironolactone and finasteride) should be avoided in the case of HT with LHRH analogs. In the case of HT with AI or SERMs, they could be used in reserve, even if the authors discourage their use until their safety is demonstrated.

### Non-hormonal agents

If it is accepted that hormonal agents are best not employed, other molecules can be considered.

Topical minoxidil 2% and 5% is commonly utilized. It induces vasodilatation by opening potassium channels localized on smooth muscle cells of peripheral arteria, slowing down circulation. Minoxidil, in addition, induces angiogenesis and activates prostaglandin-endoperoxides synthase 1 which stimulates hair growth [18]. The final minoxidil effect is to extend the anagen phase. The association with topical hydrocortisone could help follicular growth.

Freites-Martinez et al. [11] reported a moderate to significant improvement in alopecia in 80% of patients using topical minoxidil.

Bimatoprost 0.03%, a prostaglandin analog, could also be utilized [19]. It protects follicles in the anagen phase and improves follicular growth in anagen I. For these reasons, it should be utilized with the same indications of minoxidil. In addition, the costs and the availability of the molecule should be considered.

Minoxidil and prostaglandin analogs are not recommended in preventing alopecia induced by cytotoxic agents [20], but in the case of HT, they could be useful. Probably because, differently from classic chemotherapeutic drugs, HT does not have a direct toxic effect on hair follicles, but it creates an unfavorable condition for hair growth. Moreover, even if minoxidil and prostaglandin analog increases drug concentration around the hair follicle, it is possible that beyond a certain concentration, HT molecules are not able to bind their receptor sites over more, because receptor saturation would be reached.

Lastly, another promising molecule is cetirizine. It is an antihistamine anti-H1 receptor, which is able to increase prostaglandin prostaglandine E (PGE) and prostaglandine F2alpha (PGFα) synthesis, with an action similar to bimatoprost, and to reduce inflammation and prostaglandin PGD2 synthesis. This action seems to be independent of its antihistamine one. Available studies evaluated cetirizine efficacy for the treatment of AGA and other types of alopecia with good results. Recently, it has also been proposed for the treatment of alopecia induced by palbociclib [21]. For this reason and the absence of hormonal influence, cetirizine could be a good option for alopecia induced by HT [22].

### Our proposal

In light of what is described above, we propose the use of topical formulations containing minoxidil 2%, cetirizine 0.5%, and hydrocortisone butyrate in alcohol, applied once a day alongside HT duration or minoxidil 5% + hydrocortisone butyrate 0.1% in the most severe cases and/or when a previous FAGA was present.

## Conclusions

Pseudo-FAGA induced by HT for breast cancer is probably underreported if we consider the prevalence of this tumor and the common use of these therapies. Maybe because oncologists are not interested in the diagnosis of alopecia, and patients do not refer to dermatologists or they refer only if they had a previous FAGA that worsen after HT.

Anyway, even if this pattern of alopecia is similar to FAGA, its pathogenetic mechanism and management are different, so it has to be considered an oncotherapy-induced alopecia.

For this reason, and for its direct or indirect oncogenic potential, topical hormones should not be considered for the treatment of alopecia induced by HT.

With this work, we propose a therapeutic approach that could be both safe and effective in order to reduce hair miniaturization and stimulate hair growth even when a previous FAGA was present.

## Abbreviations

AI: aromatase inhibitors
DHT: dehydrotestosterone
FAGA: female androgenetic alopecia
HT: hormonal therapy
LHRH: luteinizing hormone-releasing hormone
PGD2: prostaglandin D2
SERM: selective estrogen receptor modulators

## Declarations

### Copyright

© The Author(s) 2021.
